# Supplementary material for: Drug repurposing for aging research using model organisms
Source: Aging Cell. 2017 Jun 16;16(5):1006–15. doi: 10.1111/acel.12626 (PMC5595691; doi:10.1111/acel.12626)
Supplement: Supplementary file 7 — Data S1 Zip‐Archive of all report cards. [file ACEL-16-1006-s007.zip › RC_8I1.pdf]

8I1

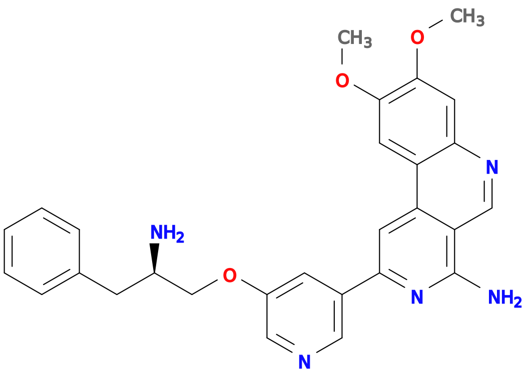

Database identifiers

ChEMBLCompound CHEMBL1095294

Ranking

	Rank	Score
Drosophila	625/697	0.141
C. elegans	489/591	0.081

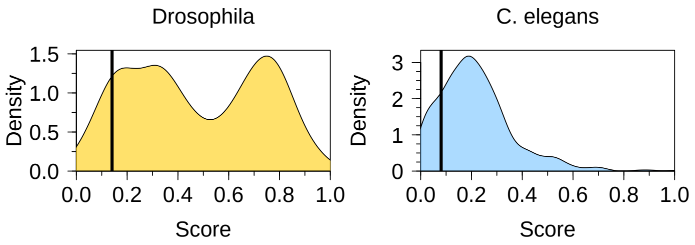

	Ageing implication	Domain conservation	Binding site conservation	Binding affinity	Bioavailability	Lipinski	Promiscuity	Purchasability	Drug approval	**Total**
Drosophila	0.36	0.696	0.904	0.937	(0.9)	-0.05	-0.0	0.0	0.0	0.141
C. elegans	0.36	0.644	0.818	0.937	0.737	-0.05	-0.0	0.0	0.0	0.081

Names

No synonyms found

Roles

ChEBI entry None has no roles

Status

Approved drug (according to ChEMBL)	No
Number of Rule of 5 violations	1
Binding affinity to original target in log units (RF-Score prediction)	7.7
Burns *C. elegans* bioavailability prediction	5.6

Compound Target Characteristics

3-phosphoinositide-dependent protein kinase 1

Best gene implication in ageing for this target family came from gene Q9Y1J3 via mapping the annotation from Ensembl WBGene00003965 via mapping the annotation from EntrezGene 180475 via mapping the annotation from GenAgeModels 0499 annotated in GenAge release 17.

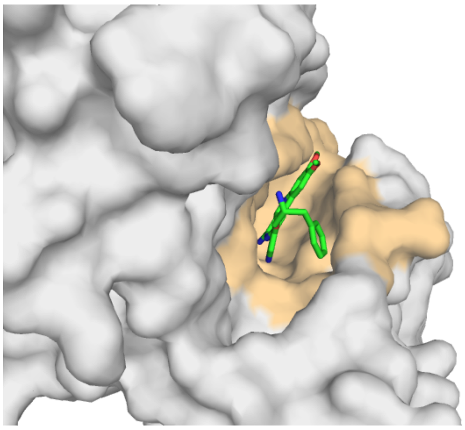

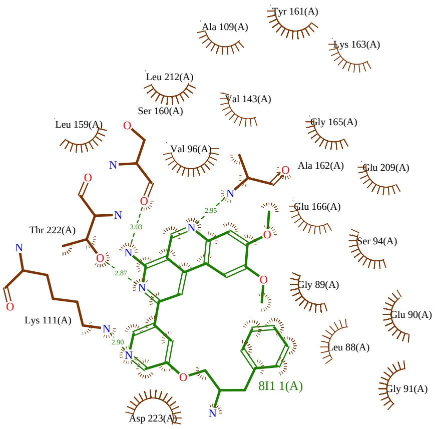

protein	amino acids contacts (binding site)																				
PDB:3iop:chainA:O15530	L	G	E	G	S	V	A	K	V	L	S	Y	A	K	G	E	E	N	L	T	D
tr:Q9UPJ8:Q9UPJ8_HUMAN	L	G	E	G	S	V	A	K	V	L	S	Y	A	K	G	E	E	N	L	T	D
tr:E9PER6:E9PER6_HUMAN	L	G	E	G	S	V	A	K	V	L	S	Y	A	K	G	E	E	N	L	T	D
tr:C9JWR9:C9JWR9_HUMAN	L	G	E	G	S	V	A	K	V	L	S	Y	A	K	G	E	E	N	L	T	D
sp:O15530:PDPK1_HUMAN	L	G	E	G	S	V	A	K	V	L	S	Y	A	K	G	E	E	N	L	T	D
sp:O55173:PDPK1_RAT	L	G	E	G	S	V	A	K	V	L	S	Y	A	K	G	E	E	N	L	T	D
tr:Q3UEW8:Q3UEW8_MOUSE	L	G	E	G	S	V	A	K	V	L	S	Y	A	K	G	E	E	N	L	T	D
tr:F2Z400:F2Z400_MOUSE	L	G	E	G	S	V	A	K	V	L	S	Y	A	K	G	E	E	N	L	T	D
tr:Q3TRL2:Q3TRL2_MOUSE	L	G	E	G	S	V	A	K	V	L	S	Y	A	K	G	E	E	N	L	T	D
tr:F2Z3X6:F2Z3X6_MOUSE	L	G	E	G	S	V	A	K	V	L	S	Y	A	K	G	E	E	N	L	T	D
sp:Q9Z2A0:PDPK1_MOUSE	L	G	E	G	S	V	A	K	V	L	S	Y	A	K	G	E	E	N	L	T	D
tr:Q810Z4:Q810Z4_MOUSE	L	G	E	G	S	V	A	K	V	L	S	Y	A	K	G	E	E	N	L	T	D
tr:A4V164:A4V164_DROME	I	G	E	G	S	V	A	K	V	M	T	Y	A	R	G	D	E	N	L	A	D
tr:C7LAC8:C7LAC8_DROME	I	G	E	G	S	V	A	K	V	M	T	Y	A	R	G	D	E	N	L	A	D
tr:H5V8B9:H5V8B9_DROME	I	G	E	G	S	V	A	K	V	M	T	Y	A	R	G	D	E	N	L	A	D
sp:Q9W0V1:PDPK1_DROME	I	G	E	G	S	V	A	K	V	M	T	Y	A	R	G	D	E	N	L	A	D
sp:Q9Y1J3:PDPK1_CAEEL	M	G	E	G	S	V	A	K	T	I	G	L	V	E	G	D	D	N	L	T	D
sp:Q03407:PKH1_YEAST	L	G	D	G	S	V	A	K	F	L	E	Y	A	P	G	D	E	N	L	T	D
sp:Q03306:PKH3_YEAST	L	G	H	G	S	V	A	K	I	L	D	F	A	P	G	E	E	N	L	T	D
sp:Q12236:PKH2_YEAST	I	G	D	G	S	V	A	K	V	L	E	Y	A	P	G	D	E	N	L	T	D

protein	whole protein ident	simil	domain-based ident	simil	contact-based ident	simil
PDB:3iop:chainA:O15530	1.0	1.0	1.0	1.0	1.0	1.0
tr:Q9UPJ8:Q9UPJ8_HUMAN	0.66	0.66	1.0	1.0	1.0	1.0
tr:E9PER6:E9PER6_HUMAN	0.95	0.95	1.0	1.0	1.0	1.0
tr:C9JWR9:C9JWR9_HUMAN	0.95	0.96	0.91	0.91	1.0	1.0
sp:O15530:PDPK1_HUMAN	1.0	1.0	1.0	1.0	1.0	1.0
sp:O55173:PDPK1_RAT	0.95	0.98	0.99	1.0	1.0	1.0
tr:Q3UEW8:Q3UEW8_MOUSE	0.73	0.76	0.99	1.0	1.0	1.0
tr:F2Z400:F2Z400_MOUSE	0.75	0.79	0.99	1.0	1.0	1.0
tr:Q3TRL2:Q3TRL2_MOUSE	0.88	0.91	0.99	1.0	1.0	1.0
tr:F2Z3X6:F2Z3X6_MOUSE	0.9	0.93	0.99	1.0	1.0	1.0
sp:Q9Z2A0:PDPK1_MOUSE	0.95	0.98	0.99	1.0	1.0	1.0
tr:Q810Z4:Q810Z4_MOUSE	0.93	0.97	0.99	1.0	1.0	1.0
tr:A4V164:A4V164_DROME	0.31	0.56	0.43	0.68	0.71	0.9
tr:C7LAC8:C7LAC8_DROME	0.28	0.5	0.43	0.68	0.71	0.9
tr:H5V8B9:H5V8B9_DROME	0.28	0.5	0.43	0.68	0.71	0.9
sp:Q9W0V1:PDPK1_DROME	0.28	0.5	0.43	0.68	0.71	0.9
sp:Q9Y1J3:PDPK1_CAEEL	0.28	0.59	0.33	0.66	0.57	0.82
sp:Q03407:PKH1_YEAST	0.21	0.47	0.44	0.74	0.76	0.85
sp:Q03306:PKH3_YEAST	0.15	0.35	0.4	0.69	0.76	0.88
sp:Q12236:PKH2_YEAST	0.15	0.35	0.44	0.75	0.76	0.87

Pdk1 (FBgn0020386) associated phenotypes

cell autonomous, chemical conditional, chemical resistant, chemical sensitive, decreased cell size, developmental rate defective, increased cell death, increased cell size, male limited, neuroanatomy defective, nutrition conditional, partially lethal - majority die, small body, somatic clone

(Information from FlyBase)

Pdk1 (UniProt:Q9W0V1) annotation

Function: Serine/threonine kinase required for embryonic development. Inhibits apoptosis. Acts in the insulin receptor transduction pathway which regulates cell growth and organ size, by phosphorylating and activating Akt1 and S6k. May be involved in axonal pathfinding and synaptogenesis, and in spermatogenesis. (PubMed:10336630, PubMed:11301252, PubMed:11344272, PubMed:11752451, PubMed:11862217, PubMed:15238523, PubMed:9368760).
Subcellular location: Cytoplasm
Developmental stage: Highly expressed in Malpighian tubule primordia from stage late 11 to 13. Expressed in hindgut at stage 13. After stage 14, ubiquitously expressed at low levels. (PubMed:11301252).
Domain: The PIF-pocket is a small lobe in the catalytic domain required by the enzyme for the binding to the hydrophobic motif of its substrates. It is an allosteric regulatory site that can accommodate small compounds acting as allosteric inhibitors. (UniProtKB:O15530).
Disruption phenotype: Death during the second instar stage. (PubMed:11752451).

(Information from UniProt)

pdk-1 (WBGene00003965) associated phenotypes

DMPP resistant, carbon dioxide avoidance variant, dauer constitutive, dauer recovery inhibited, egg laying variant, extended life span, long, reduced brood size, shortened life span, social feeding increased

(Information from WormBase)

pdk-1 (UniProt:Q9Y1J3) annotation

Function: Involved in the daf-2/insulin receptor-like transduction pathway, which controls longevity and prevents developmental arrest at the dauer stage. Phosphorylates and activates sgk-1, akt-1 and akt-2. (PubMed:10364160, PubMed:15068796).
Subunit: Interacts directly with sgk-1, akt-1 and akt-2.(PubMed:15068796).
Subcellular location: Cytoplasm
Developmental stage: Expressed in late stage embryos and throughout life. At L1, expressed

in neurons, intestinal cells and hypodermal cells. In adults, expressed in the somatic gonad. (PubMed:10364160).

Domain: The PIF-pocket is a small lobe in the catalytic domain required by the enzyme for the binding to the hydrophobic motif of its substrates. It is an allosteric regulatory site that can accommodate small compounds acting as allosteric inhibitors. (UniProtKB:O15530).

(Information from UniProt)
